# Cerebrospinal fluid proteomics reveals the innate immunity and blood-brain barrier dysregulation in a patient with multidrug-resistant *Acinetobacter baumannii* ventriculitis treated with intrathecal and intravenous polymyxin B

**DOI:** 10.1016/j.heliyon.2024.e40893

**Published:** 2024-12-07

**Authors:** Mengyao Li, Dongyu Liu, Phillip J. Bergen, Silin Liang, Juan Chen, Zhi Ying Kho, Jing Lu, Huiying Sun, Weiqing Hong, Xiaofen Liu, Chengying Hong, Youlian Chen, Wei Li, Hongxia You, Shunyao Xu, Yu Wang, Huaiji Gao, Chun Hin Lam, Jian Li, Xiaoyin Chen, Xueyan Liu

**Affiliations:** a Department of Critical Care Medicine, The Second Clinical Medical College, Jinan University (Shenzhen People's Hospital), Shenzhen, 518020, China; b Integrated Chinese and Western Medicine Postdoctoral Research Station, Jinan University, Guangzhou, 510632, China; c Biomedicine Discovery Institute, Infection Program and Department of Microbiology, Monash University, Melbourne, VIC, 3800, Australia; d Department of Pharmacy, The Second Clinical Medical College, Jinan University (Shenzhen People's Hospital), Shenzhen, 518020, China; e Institute of Infectious Diseases, The Second Hospital of Tianjin Medical University, 23 Pingjiang Road, Tianjin, 300211, China; f Institute of Antibiotics, Huashan Hospital, Fudan University / Key Laboratory of Clinical Pharmacology of Antibiotics, National Health Commission of the People's Republic of China, Shanghai 200040, China; g National Clinical Research Centre for Aging and Medicine, Huashan Hospital affiliated to Fudan University, Shanghai, 200040, China; h Department of Stomatology, The Second Clinical Medical College, Jinan University (Shenzhen People's Hospital), Shenzhen, 518020, China; i Mathematics and Statistics, School of Computing Engineering and Mathematical Sciences, La Trobe University, Melbourne, VIC, 3085, Australia; j Faculty of Medicine, Macau University of Science and Technology, Taipa, Macau, 999078, China; k College of Traditional Chinese Medicine, Jinan University, Guangzhou, 510632, China

**Keywords:** Cerebrospinal fluid, Proteomics, *Acinetobacter baumannii*, Ventriculitis, Polymyxin B, Intrathecal and intravenous administration

## Abstract

*Acinetobacter baumannii* is a major pathogen of nosocomial meningitis and ventriculitis. Due to very limited antibiotic treatment options, polymyxins are often used as a last-line therapy. To optimise polymyxin use in the intraventricular environment, cerebrospinal fluid (CSF) proteomics was employed to investigate host-pathogen-polymyxin interactions in a 69-year-old patient with multidrug-resistant *A. baumannii* ventriculitis treated with a combination of intrathecal (ITH; 50,000 IU q24h/q48h), intraventricular (IVT; 50,000 IU q48h), and intravenous (500,000 IU, q12h) polymyxin B. CSF was collected before the first ITH dose in the ICU (0 h) and at 24 h, Day 7 and Day 26. The proteome was quantified at each time point and proteins with Qvalue <0.05 and fold change >1.2 were considered differentially expressed. Within 24 h of ITH/IVT polymyxin B administration, the innate immune system and neuroimmunity were highly active, evidenced by up-regulation of various pathways related to pathogen invasion, endocytosis and neutrophil degranulation. Blood-brain barrier impairment had worsened at 24 h but signs of repair were evident on Day 7 and Day 26. This is the first CSF proteomic study with polymyxins. Our findings provide critical mechanistic insights into optimizing ITH/IVT polymyxin administration.

## 1 Introduction

Multidrug-resistant (MDR) *Acinetobacter baumannii* is a major nosocomial pathogen in patients who are immunosuppressed or have serious underlying diseases [[1], [2], [3]]. MDR *A. baumannii*, including carbapenem-resistant strains (CRAB), can cause ventilator-associated pneumonia, bacteraemia, and infections of the urinary tract, skin, soft tissue, and central nervous system (CNS) [[4], [5], [6]]. Notably, *A. baumannii* is responsible for approximately 11 % of nosocomial meningitis and ventriculitis, with mortality ranging from 15 % to 70 % [[7], [8], [9]]. Treating CRAB is challenging because once *A. baumannii* exhibits carbapenem resistance, it has typically acquired resistance to most antibiotics that would otherwise be effective against wild-type *A. baumannii* [10]. Currently, high-dose ampicillin-sulbactam (total daily dose of 6–9 g of the sulbactam component), combined with other antibiotics such as polymyxins, minocycline, tigecycline, and cefiderocol, is recommended [10]. Meningitis and ventriculitis caused by MDR *A. baumannii* are particularly difficult to treat given the poor penetration of many antibiotics across the blood-brain barrier (BBB) following oral and intravenous administration [11,12].

Due to limited available therapeutic options, the ‘old’ polymyxins have become a last-line treatment for infections caused by MDR *A. baumannii* [13,14]. Polymyxins were first discovered in 1947 and approved in the late 1950s but were abandoned in the late 1970s due to toxicities [15]. By the mid-1990s, polymyxins were reintroduced into the clinic because of the rapid emergence of drug-resistant Gram-negative bacteria resistant to all other therapeutic options [16]. Polymyxins are cyclic lipopeptides that retain significant *in vitro* activity against many problematic Gram-negative pathogens, including *A. baumannii* [17,18]. Two polymyxins, namely polymyxins B and E (the latter known as colistin), are available in clinical practice [19]. Only in China is colistin commercially available as colistin sulfate (the antibacterial entity) for oral and topical use, as well as for intravenous administration. In China and elsewhere, colistin is commonly available for parenteral administration in the form of an inactive prodrug, sodium colistin methanesulphonate (CMS; aka colistimethate sodium), which requires conversion to the active entity, colistin *in vivo* [20]. Polymyxin B is available globally except in Europe and only comes as the active form, polymyxin B sulfate. Both polymyxins can be administered intrathecally (ITH) and intraventricularly (IVT) for treatment of CNS infection [21].

The transport of polymyxins across a healthy blood-brain barrier (BBB) is negligible and remains very low in patients with systemic inflammation [22,23]. Consequently, primary or adjunct ITH or IVT administration of polymyxins to bypass the BBB is commonly used in clinical practice to treat CNS infections caused by MDR Gram-negative pathogens [24]. Currently available clinical data on the use of ITH/IVH polymyxins is sparse but suggests that substantially higher polymyxin concentrations are achieved in the cerebrospinal fluid (CSF) following ITH or IVT administration compared to intravenous administration [24]. The currently recommended ITH and IVT daily doses of 5 mg (50,000 international units [IU]) for polymyxin B and 125,000 IU (∼4.1 mg colistin base activity [CBA]) for CMS are largely empirical [21]. Previous studies on the ITH or IVT administration of polymyxins have primarily consisted of clinical case series observing the diagnosis, treatment, and outcome of patients [[25], [26], [27], [28]]. There is an urgent need for research into the mode of action, as well as the pharmacokinetics, pharmacodynamics, and toxicodynamics (PK/PD/TD) of polymyxins administered via ITH or IVT, to optimise dosage regimens.

The special micro-environment of the CNS is protected and maintained by the BBB, which consists mainly of a basement membrane and a monolayer of tightly-sealed microvascular endothelial cells, resulting in low paracellular and transcellular permeability [29]. CNS infections can increase the permeability of the BBB and lymphocyte trafficking, thereby promoting the influx of innate immune cells [30]. Polymyxins may also impact the BBB. To optimise the use of ITH/IVT polymyxins for treating CNS infections, it is essential to examine the interactions among the host, pathogen, and polymyxins. CSF is secreted by various CNS structures and is primarily composed of water (99 %), with the remaining 1 % consisting of proteins, ions, neurotransmitters, and glucose [31]. Changes in CSF composition accurately reflect pathological processes in the CNS. Proteomics offers a comprehensive view for analysing CSF proteins at a systems level [32].

The aim of this study was to investigate the interactions among the host, pathogen, and polymyxins after ITH/IVT administration at the systems protein level. It is the first study to employ CSF proteomics to investigate these interactions in a patient with MDR *A. baumannii* ventriculitis treated with ITH and IVT polymyxin B. The proteomics analysis revealed that the innate immune system and neuroimmunity were highly active within 24 h of ITH/IVT polymyxin B administration, BBB impairment worsened at 24 h, but signs of repair were evident on Day 7 and Day 26.

## 2 Materials and methods

### 2.1 Patient case history

The study was conducted in accordance with the principles of the Helsinki Declaration and was approved by the Ethic Committee of Shenzhen People's Hospital, Shenzhen, China (Approval No. LL-KY-2021620). Informed consent was obtained from the patient's family. The inclusion and exclusion criteria were as follows: (a) patients aged 18 years or older; (b) CSF cultures indicated MDR *A. baumannii*; (c) meeting the diagnostic criteria for intracranial infection [33]; (d) patients treated with ITH and/or IVT injection of polymyxins. Pregnant or lactating females were excluded for physiological and ethical reasons [34].

**Fig. 1:**
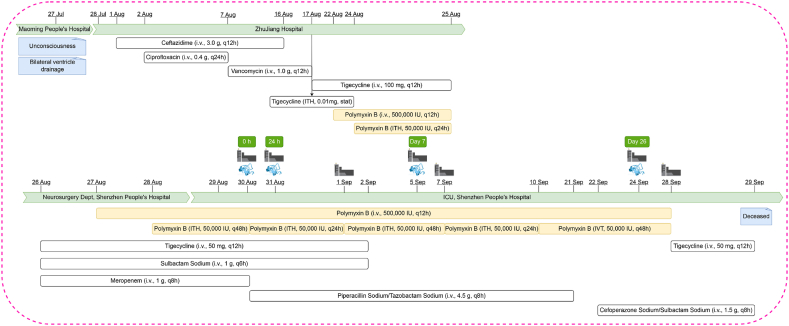
Patient history. The blue protein icons show the days on which CSF samples were taken for proteomic analysis, the grey machine icons show the days on which TDM was conducted.

An unconscious 69-year-old female presented to Maoming People's Hospital (Maoming, Guangdong Province) on July 27th, 2021 with trauma-induced paraplegia since 1985 and a history of hypertension (>7 years; no regular medications) (Fig. 1). A computed tomography (CT) scan of her head and chest showed bleeding of the left basal ganglia into the left cerebral ventricle and infections in both lungs. Bilateral external ventricular drainage was performed under general anesthesia, and she was transferred to ZhuJiang Hospital in Guangzhou on July 28th, 2021. A follow-up CT scan indicated her bleeding was much improved but her lung infection had worsened. Over the next three weeks, various antibiotics were administered intravenously (ceftriaxone, ciprofloxacin, vancomycin) and intrathecally (tigecycline), either alone or in combination. On August 15th, 2021, lumbar drainage was performed. On August 22nd, 2021, a CT scan of her head and chest showed reduced ganglia and ventricular bleeding and significant absorption of subarachnoid hemorrhage. Pneumatosis under the skull plate on the right forehead was significantly reduced. Pneumonia in the lower lobes of both lungs with partial consolidation had progressed with a small amount of bilateral pleural effusion. An MDR strain of *A. baumannii* was isolated and identified from both CSF and sputum cultures. CSF biochemistry revealed the following: chlorine, 117.4 mmol/L; glucose, 2.17 mmol/L; total cell number, 23871/μL; total protein, 0.77 g/L; positive for globulin; and nucleated cell count, 13871/μL. To the ongoing ITH and IV tigecycline regimen, IV polymyxin B was added due to the identification of MDR *A. baumannii* from the CSF culture which was only susceptible to tigecycline and polymyxins. On August 24th and 25th, 2021, lumbar punctures were performed and ITH polymyxin B was administered. On August 26th, 2021, the patient was transferred to the Neurosurgery Department of Shenzhen People's Hospital where IV tigecycline, sulbactam and meropenem were commenced. The following day, IV polymyxin B was added to this regimen, with ITH administration added the next day. On August 29th, 2021, the patient was transferred to the intensive care unit (ICU) where her current antibiotic regimens of polymyxin B, tigecycline, sulbactam sodium and meropenem were continued. Over the next month, IV administration of polymyxin B continued which was supplemented with either ITH or intraventricular (IVT) polymyxin B. However, all polymyxin administration was paused on September 28th, 2021 due to decreased kidney function, and tigecycline commenced. Unfortunately, the patient died of severe sepsis the next day. All antibiotic regimens (dose and duration) are shown in Fig. 1.

Clinical indicators were measured periodically throughout the patient's admission to evaluate systemic and CNS inflammation and kidney function. Four common plasma biomarkers, namely C-reactive protein, interleukin 6 (IL-6), white blood cell count, and procalcitonin were used to assess systemic inflammation.

### 2.2 CSF samples for therapeutic drug monitoring (TDM) and proteomic analysis

In the ICU, CSF samples were obtained from the patient at 5 min before each ITH administration via lumbar puncture or the ventricle drainage tube for measurements of the concentration and potential toxicity of polymyxin B. CSF was collected immediately before the first ITH dose administered in the ICU (0 h) and immediately before the doses at 24 h, 7 days, and 26 days (shown by the green boxes in Fig. 1). Samples were promptly centrifuged (2000×*g*, 10 min, 4 °C) to remove cells, and 1 mL aliquots of the supernatant were transferred to cryovials prior to storage at −80 °C [35]. At the same times, blood samples were collected in heparin anticoagulant tubes, immediately centrifuged (2000×*g*, 10 min, 4 °C), and the supernatant stored at −80 °C [36]. Supernatant samples were delivered with dry ice to Huashan Hospital for therapeutic drug monitoring and to BGI Genomics (Shenzhen, China) for proteomic experiments. The concentration of LPS was quantified by Limulus Amebocyte Lysate (LAL) assay (Beyotime, Shanghai, China).

### 2.3 TDM of polymyxin B in CSF and plasma

TDM was performed for polymyxin B in both CSF and plasma using our previously developed liquid chromatography-tandem mass spectrometry (LC-MS/MS) method [37,38]. The reference standard of polymyxin B was obtained from the USP (batch no. R046V0, Rockville, USA) and in every 1 mg of polymyxin B contained 0.734 mg polymyxin B1, 0.086 mg B1-Ile and 0.090 mg polymyxin B2. Polymyxin E2 (purity: 96.2 %) was obtained from the SPH No. 1 Biochemical & Pharmaceutical Co. Ltd (Shanghai, China) was used as the internal standard.

### 2.4 Proteomics analysis

Tandem mass tag (TMT)-based proteomics, which offers advantages in terms of high proteome coverage, precise and accurate quantification, and can accommodate to 18 multiplexed samples in a single experiment, was used to quantify proteins in CSF [39]. All TMT mass spectrometry experiments were conducted by BGI Genomics. Proteins were extracted from CSF samples with cold acetone and dithiothreitol (DTT; final concentration of 10 mM). The quality of extraction was measured by the Bradford method followed by sodium dodecyl sulfate polyacrylamide gel electrophoresis (SDS-PAGE) [40]. Proteins were then digested with trypsin in an enzyme to substrate ratio of 1:20 (μg/μg), centrifuged at 10,621 *g* for 1 min, and incubated at 37 °C for 4 h. Each tube of TMT reagent (0.8 mg) was dissolved in 41 μL acetonitrile (ACN). Digested peptides were dissolved in 0.1 M triethylamonium bicarbonate (TEAB) buffer to a final peptide concentration of 3.74 μg/μL. Subsequently, 100 μg (26.7 μL) of peptides and 41 μL of TMT reagent were thoroughly mixed and centrifuged, resulting in a final solution with a pH of between 7.0 and 8.0. The TMT-labeled peptides were fractionated for mass spectrometry (MS) analysis by a Shimadzu LC-20AB liquid phase system. The dried peptide samples were reconstituted with mobile phase A (5 % ACN, pH 9.8) and eluted by a multi-step gradient system at a flow rate of 1 mL/min. Elution began with 95 % mobile phase A and 5 % mobile phase B (95 % ACN, pH 9.8) for 10 min, 65 % mobile phase A and 35 % mobile phase B for 40 min, 5 % mobile phase A and 95 % mobile phase B for 1 min, 100 % mobile phase B for 3 min, finishing with 95 % mobile phase A and 5 % mobile phase B for 10 min. Samples were freeze-dried after collection of the elution peak (wavelength, 214 nm). The dried peptides were reconstituted with mobile phase A (2 % ACN, 0.1 % formic acid [FA]) and separated by high-performance liquid chromatography (HPLC) using an Easy-nLC 1200 system (Thermo Fisher Scientific, San Jose, CA) with a tandem self-packed C18 column. Separation was conducted at a flow rate of 200 nL/min using the following multi-step gradient system: 95 % mobile phase A and 5 % mobile phase B (80 % ACN, 0.1 % FA) for 3 min; mobile phase B was then linearly increased from 8 % to 44 % from 3 to 45 min, from 44 % to 60 % from 45 to 50 min, and from 60 % to 100 % from 50 to 53 min. Mobile phase B was then linearly decreased to 80 % from 53 to 60 min. After separation, the peptides were passed into an Oritrap Exploris 480 tandem mass spectrometer (Thermo Fisher Scientific, San Jose, CA).

The MS/MS spectra were analysed using Mascot software [41], with searches performed against the Swiss-Prot human database from the UniProt Knowledgebase [42]. IQuant was used to quantify proteins and identify differentially expressed proteins (DEPs) [43]. To assess peptide confidence, peptide-spectrum matches (PSMs) were pre-filtered to achieve a PSM-level FDR of ≤1 %. Identified peptides were then assembled into a series of confident proteins based on the principle parsimony [44]. To control the false positive rate at the protein level, a protein-level FDR of ≤1 % was established using the ‘picked’ protein FDR strategy [45].

### 2.5 Bioinformatic analysis

A protein with Qvalue <0.05 and fold change >1.2 was considered differentially expressed. Reactome and KEGG pathway enrichment was conducted with WebGestalt [46] and visualised by R package ggplot2 [47]. Innate immunity proteins were annotated with InnateDB, a knowledgebase of innate immunity networks, pathways, and genes [48].

## 3 Results

### 3.1 Therapeutic drug monitoring and clinical indicators

**Fig. 2:**
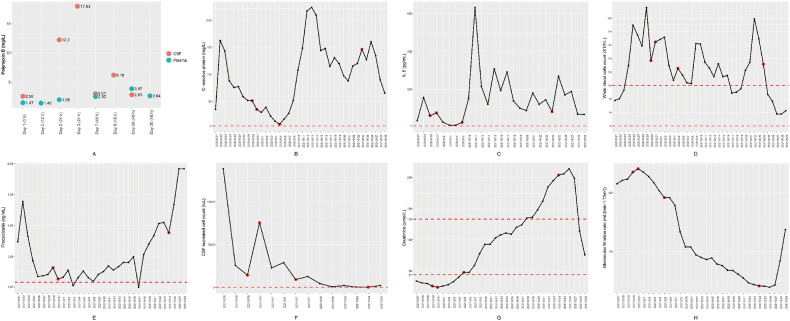
Polymyxin B concentrations and clinical indicators evaluating the patient's systemic and CNS inflammation and kidney function. A) Polymyxin B concentrations in plasma and CSF during the patient's ICU stay, the hours after each ITH/IVT dose are indicated in the brackets; B-E) examined systemic inflammation: B) C-reactive protein (normal <5 mg/L); C) IL-6 (normal <7 pg/mL); D) white blood cells count (4 <normal <10 × 10^9^/L); E) procalcitonin (normal <0.05 ng/mL). F) examined CNS inflammation using CSF nucleated cell count (normal <20/μL). G-H) examined renal function: G) plasma creatinine (44 < normal <133 μmol/L); H) glomerular filtration rate. The red dashed lines indicate normal concentrations while the red dots show measurements on days CSF proteomic samples were collected.

Neurotoxicity is an adverse effect associated with intravenous administration of polymyxins, with extended exposure as a major risk factor [49]. Therefore, therapeutic monitoring of polymyxin B concentrations in both plasma and CSF began with the first ITH administration of polymyxin B in the ICU and continued until the day before discharge. Concentrations of polymyxin B ranged from 2.58 to 17.9 mg/L in CSF over 24 h or 48 h, and 1.42–3.87 mg/L in plasma over 12 h (Fig. 2A). Though substantial fluctuations were evident, levels of C-reactive protein, IL-6, procalcitonin and the majority of white blood cells were elevated over their normal levels (Fig. 2B–E), indicating the presence of systemic bacterial infection. Inflammation in the CSF was monitored using the CSF nucleated cell count as inflammatory indicator (Fig. 2F). The gradually decreased CSF nucleated cell counts after 24 h (2021/8/31 on Fig. 2F) of the ITH polymyxin indicating the efficacy of the polymyxin B ITH administration. Creatinine concentrations and glomerular filtration rate were used to determine renal function (Fig. 2G and H). The increase of creatinine and decrease of glomerular filtration rate over time indicate nephrotoxicity.

### 3.2 Majority of CSF proteins were associated with the immune response

**Fig. 3:**
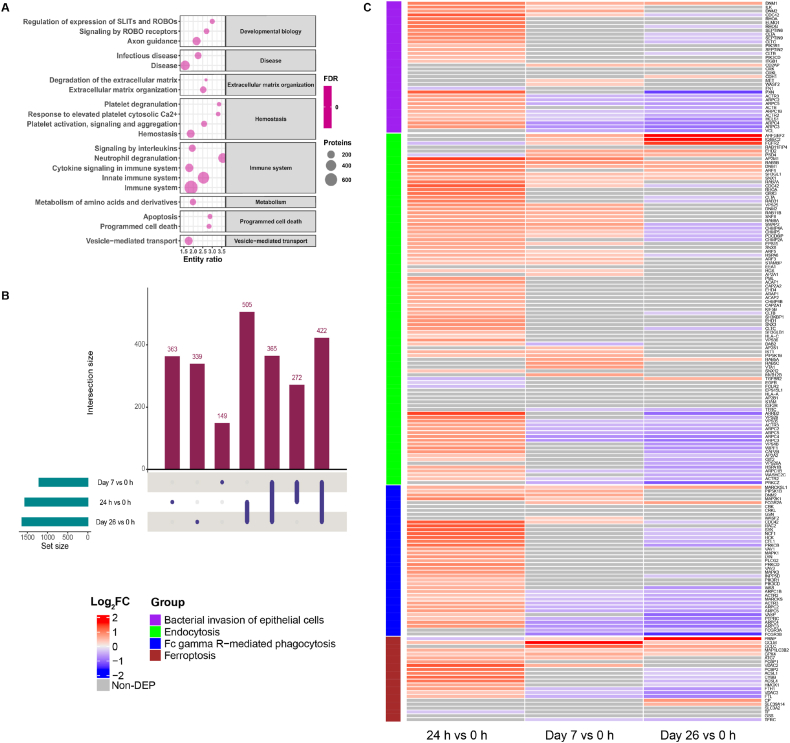
Analysis of CSF proteins and DEPs. A) Top 20 Reactome pathways enriched by the CSF proteome. The entity ratio is the proportion of Reactome pathway proteins represented by the specified pathway. B) Number of differentially expressed proteins and their distinctive intersections. C) Heatmaps for bacterial invasion and immune response pathways enriched by the 422 DEPs shared by three time points.

TMT quantification identified 2677 proteins in the CSF. Analysis with Reactome predicted the majority were associated with either the immune system (826 proteins; 30.9 %) or signal transduction (819 proteins; 30.6 %) (Fig. S1). Three additional pathways (metabolism, disease, and metabolism of proteins) also featured prominently, with each mapped to more than 600 proteins. When the false discovery rate (FDR) was set at <0.01, 339 pathways were significantly enriched (Table S1) and 82 showed an FDR very close to zero (<2.2E-16 in Reactome). The top 20 pathways encompassing the largest number of DEPs were associated with developmental biology, disease, extracellular matrix organization, hemostasis, immune system, metabolism, programmed cell death, and vesicle-mediated transport (Fig. 3A). Of these, five pathways were associated with the immune system and four with hemostasis. The immune system involved 703 proteins in this CSF proteomic profile, 500 from the innate immune system of which 319 were from neutrophil degranulation, the latter a process by which neutrophil release granules containing microbicidals.

### 3.3 Bacterial invasion pathways were activated by 24 h

At 24 h after the first ITH administration of polymyxin B in the ICU, 1562 DEPs were identified with 1277 (81.8 %) up-regulated and 285 (18.2 %) down-regulated (Fig. 3B). KEGG pathway analysis identified 12 pathways significantly perturbed with common 422 DEPs at each time (24 h *vs*. 0 h, Day 7 *vs*. 0 h, and Day 26 *vs*. 0 h; FDR <0.05, Fig. S2), which were related to bacterial invasion, immune responses, infectious disease, metabolism and BBB. Specifically, the pathways closely aligned with bacterial invasion include bacterial invasion of epithelial cells, endocytosis and Fc gamma R-mediated phagocytosis (Fig. 3C). Two bacterial uptake mechanisms associated with the bacterial invasion of epithelial cells pathway, namely the zipper and trigger models, were both activated at 24 h. In the zipper model, Arp2/3-mediated actin polymerization was triggered by the up-regulation of activators PI3K (PIK3CD and PIK3R1), Cdc42 (CDC42) and Arp2/3 (ARPC1B, ARPC2, ARPC3, ARPC4 and ARPC5), with downstream actin (ACTB) and septin (SEPTIN2, SEPTIN6 and SEPTIN9) also up-regulated (Fig. 3C). The classical extracellular cell adhesion protein fibronectin (FN1) was down-regulated 1.3-fold but its receptor integrin (ITGB1) and integrin-linked kinase (ILK) were up-regulated 1.3- and 2.0-fold, respectively. The cytoskeletal proteins dynamin (DNM1 and DNM2), cortactin (HCLS1), clathrin (CLTA, CLTB and CLTC) and paxillin (PXN) were also increased at 24 h. In the trigger model, the central regulators of the actin cytoskeleton RhoG (RHOG) and ELMO (ELMO1) were both increased 2.1-fold.

Within the endocytosis pathway at 24 h, 66 proteins were up-regulated and 5 down-regulated (Fig. 3C). As the key process of transporting a wide range of cargo molecules from the cell surface to the interior, clathrin-dependent endocytosis generates the endocytic vesicles using clathrin [50], of which two light chains (CLTA and CLTB) and one heavy chain (CLTC) were increased by 1.9-, 1.5- and 1.7-fold, respectively. However, at Day 26 these three clathrin proteins were decreased. Another essential component of the clathrin-coated vesicles is the AP-2 adaptor complex. At 24 h, two subunits of this complex were up-regulated, namely AP2M1 (2.8-fold) and AP2A2 (1.3-fold). At Day 7, three AP-2 subunits (AP2M1, AP2A1 and AP2S1) were significantly up-regulated as well.

Phagocytosis is the central component of inflammation and defense against infectious agents and is activated when the Fc gamma receptor on macrophages or neutrophils combined with IgG [51]. In the present study, the key signal transductor in the phagocytosis, spleen associated tyrosine kinase (Syk), was increased by 2.4-fold at 24 h, but decreased by 1.3-fold at Day 26. Dynamin II (DNM2) is in endomembranes and plasma membranes and is responsible for budding and scission of endocytic pits [52]; in the present study, DNM2 was up-regulated by 1.7-fold at 24 h and 1.6-fold at Day 7. Another dynamin isoform, dynamin I (DNM1), which is abundant in the brain, was significantly increased by 2.0-, 1.5-, and 1.6-fold at 24 h, Day 7 and Day 26, separately.

### 3.4 Immune responses were enhanced 24 h after ITH polymyxin administration

**Fig. 4:**
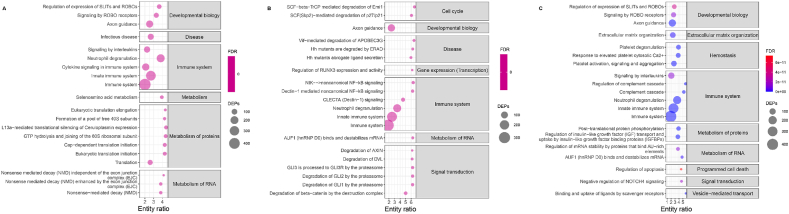
Top 20 Reactome pathways enriched with DEPs from three comparisons (A) 24 h vs. 0 h, (B) Day 7 vs. 0 h, (C) Day 26 vs. 0 h.

Using Reactome enrichment analysis, the top 20 pathways enriched with DEPs at 24 h after ITH polymyxin administration were associated with developmental biology, disease, immune system, metabolism, metabolism of proteins, and metabolism of RNA (Fig. 4A). Innate immunity and neuroimmunity were especially enhanced 24 h after the first administration of ITH polymyxin B, most notably ferroptosis and leukocyte transendothelial migration pathways. The ferroptosis pathway marker transferrin (TF) promotes this process and was down-regulated 1.2-fold at 24 h (Fig. 3C). In contrast, two ferroptosis inhibition markers, glutathione peroxidase 4 (GPX4) and ferritin (FTH1 and FTL), were significantly increased at 24 h. On Day 7 and Day 26, GPX4 remained up-regulated while ferritin heavy and light chains (FTH1 and FTL) were down-regulated.

**Fig. 5:**
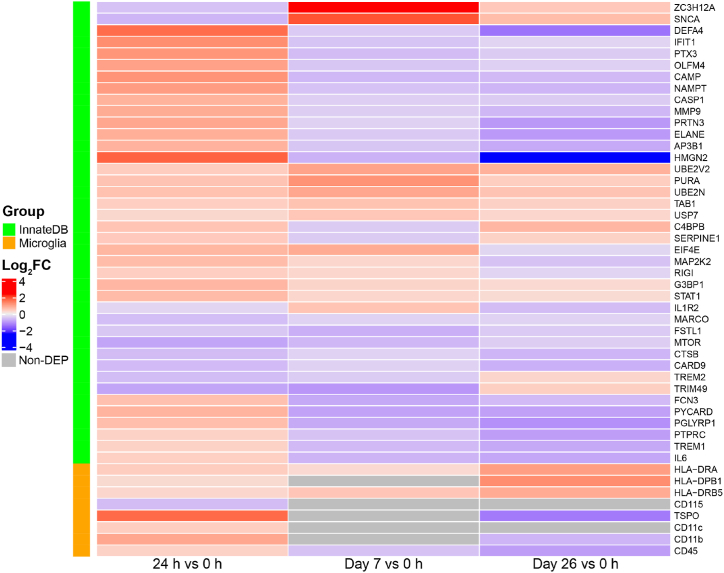
Heatmaps showing expression changes of DEPs in innate immunity and neuroimmunity at three time points. Two groups of DEPs are presented: innate immunity associated DEPs curated in InnateDB and microglia markers.

Annotated by InnateDB, 270 proteins were involved with innate immunity, with 40 significantly dysregulated at 24 h, Day 7 and Day 26 (Fig. 5). Of these 40 proteins, 30 were up-regulated at 24 h, while 27 and 27 were down-regulated at Day 7 and Day 26, respectively. Neutrophils are the most abundant leukocytes and the first innate immune cells recruited to the bacterial infection site [53]. They fulfill an antibacterial role via the process of degranulation whereby neutrophils mobilize antimicrobial proteases containing granules to fuse with cell or phagosomal membranes, resulting in the exocytosis or exposure of membrane proteins [54]. Antimicrobial peptides α-defensin 4 (DEFA4), neutrophil elastase (ELANE), cathelicidin antimicrobial peptide (CAMP) and proteinase 3 (PRTN3) contained in neutrophil granules and released upon neutrophil degranulation were elevated by 3.2-, 1.8-, 2.3- and 2.0-fold at 24 h, respectively, indicating a strong neutrophil response was triggered. Other proteins in the neutrophil degranulation were also increased at 24 h, namely pentraxin 3 (PTX3), PYD and CARD domain containing (PYCARD), peptidoglycan recognition protein 1 (PGLYRP1), matrix metallopeptidase 9 (MMP9) and olfactomedin 4 (OLFM4). Neuroimmunity was also activated at 24 h and changes in expression of markers for microglia are shown in Fig. 5.

BBB impairment worsened 24 h after ITH polymyxin administration but was improving at Day 7 and Day 26.

**Fig. 6:**
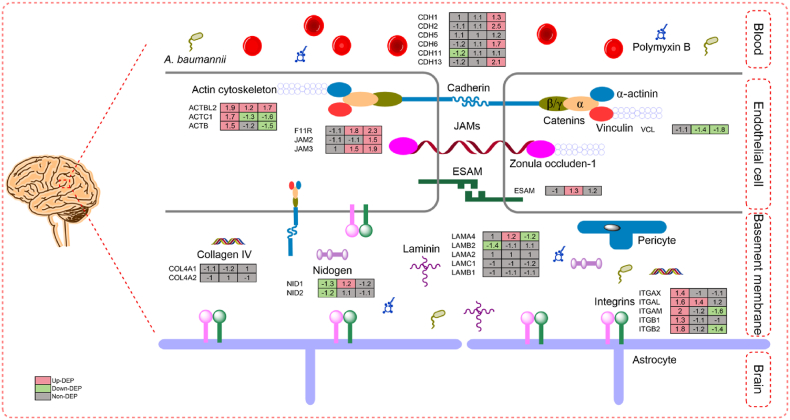
Perturbations of the BBB after ITH administration of polymyxin B. Fold-change values for three comparisons are denoted on the heatmaps: (left) 24 h vs 0 h, (middle) Day 7 vs 0 h, and (right) Day 26 vs 0 h.

Expression changes of DEGs associated with the BBB are shown in Fig. 6. Expression changes of junctional adhesion molecules and basement membrane components revealed impairment of the BBB 24 h after commencement of ITH polymyxin administration. This impairment was possibly due to the cerebral hemorrhage and following ventriculitis. Tight junction protein complexes are the main contributors to the physical barrier which controls the low paracellular permeability among apposing brain microvascular endothelial cells [55]. Although only cadherin 11 (CDH11) was significantly down-regulated at 24 h using our DEP thresholds, the expression of most other junctional adhesion molecules including cadherin 2, 6 and 13 (CDH2, CDH6 and CDH13), vinculin (VCL), F11R (JAM1), JAM2, and endothelial cell adhesion molecule (ESAM) was decreased (Fig. 6). These tight junction proteins attach to the cellular actin cytoskeleton which maintains BBB integrity [56]. The expression of actin increases when BBB permeability increases [57]. Not surprisingly, three actin isoforms ACTBL2, ACTC1 and ACTB were significantly up-regulated at 24 h by 1.9-, 1.7- and 1.5-fold, respectively.

The basement membrane of the BBB is an extracellular matrix of four main structural proteins: collagen IV family proteins, nidogens, laminins, and heparan sulfate proteoglycans [30,58]. At 24 h there were significant decreases in laminin subunit beta 2 (LAMB2; 1.4-fold) and nidogen 1 and 2 (NID1 and NID2; 1.3- and 1.2-fold, respectively), indicating disrupted stability and integrity of the BBB. Integrins are heterodimeric transmembrane cell adhesion molecules between the extracellular matrix and the actin cytoskeleton that also interact with signaling proteins to stimulate intracellular signaling cascades [56,59]. Interestingly, integrin subunits αX, αL, αM, β1 and β2 were all increased at 24 h (range, 1.3- to 2.0-fold). Collectively, these expression changes of BBB components strongly indicate impairment of the BBB at 24 h.

In contrast to the situation at 24 h, the aforementioned proteins were up-regulated at Day 7 and Day 26 (Fig. 6). Notably, junctional adhesion molecules responsible for BBB structural stability and integrity were widely increased at Day 7 (CDH1, CDH2, CDH6, CDH11, F11R, JAM3 and ESAM) and Day 26 (CDH1, CDH2, CDH5, CDH6, CDH11, CDH13, F11R, JAM2 and JAM3). Basement membrane proteins including LAMA4, NID1 and NID2 were also increased 1.2- to 1.2-fold at Day 7. At Day 26, the majority of junctional adhesion molecules were significantly up-regulated, including cadherin isoforms CDH1, CDH2, CDH6 and CDH13 and JAM isoforms F11R, JAM2 and JAM3. Three integrin isoforms (ITGAX, ITGAM and ITGB2) were decreased at Day 7 (by 1.1-, 1.6- and 1.4-fold, respectively), with ITGAX, ITGAM and ITGB2 also decreasing at Day 26 (by 1.1-, 1.6- and 1.4-fold). This suggests that repair of the BBB occurred as early as Day 7 and continued till Day 26 at least.

## 4 Discussion

Polymyxins are increasingly used to treat CNS infections caused by MDR Gram-negative bacteria [24]. However, there is a significant lack of clinical data on ITH and IVT administration of polymyxins, and as a result, current dosing regimens are empirical and not yet optimized. Our study is the first CSF proteomic study to examine responses to ITH/IVT administration of polymyxins in a critically-ill patient with ventriculitis. The CSF proteins dysregulated by ITH/IVT administered polymyxin B were primarily associated with innate immunity, with most proteins significantly increased at 24 h following polymyxin B administration. Notably, the BBB was impaired at 24 h, but signs of repair were obvious at Day 7 and afterwards. These temporal changes indicated the prominent antimicrobial activity of ITH/IVT polymyxin B and the host response at 24 h, as well as distinct BBB repair activity of polymyxin B. These findings provide a comprehensive view of the host-pathogen-polymyxin interactions in the context of ITH/IVT administration.

The patient had a long history of hypertension without regular medications, which may have contributed to the cerebral hemorrhage. *A. baumannii* ventriculitis and subsequent systemic infection were the major complications of external ventricular drainage [60]. Ultimately, the patient died from sepsis, which involved failures in respiratory system, coagulation, liver, and kidneys. *A. baumannii* was not isolated from the patient's CSF culture during her ICU stay, very likely due to the antibiotic treatment during her previous hospital stay. Nevertheless, empirical treatment with polymyxin B started shortly after ICU admission (i.e. 0 h in this study) based on the epidemiological data of antibiotic resistance in our hospital [61]. The reduced levels of CSF inflammatory biomarkers across this time indicated the efficacy of polymyxin B treatment in curing potential bacterial infection in the CSF. Therefore, 24–48 hourly administration of ITH/IVT polymyxin B continued throughout the patient's ICU admission.

In the literature on ITH or IVT administration of polymyxin B in patients, the most common dosage regimen is 5 mg/24 h and the duration of administration typically ranges from 1 to 9 weeks [23,24]. In the present study, the patient was administered ITH/IVT polymyxin B intermittently for approximately 5 weeks (Fig. 1). Considering the safety of the patient, concentrations of polymyxin B in CSF were monitored from Day 1. Although the plasma concentrations ranged from 1.42 mg/L to 3.87 mg/L, the CSF concentrations reached as high as 17.9 mg/L on Day 3, which are well above the recommended susceptibility breakpoint of 2 mg/L for most Gram-negative pathogens [62]. Such a high concentration may be attributable to the accumulation of polymyxins in the CSF due to low clearance. In the literature, even higher concentrations of polymyxin B in the CSF (29.4 mg/L) were reported after ITH/IVT administration (50,000 U/day for 4 days, then 50,000 U/2 days plus IV polymyxin B at 450,000 U/12 h) in a 14-year-old male meningitis patient infected by *A. baumannii* [63].

Nephrotoxicity was observed on September 28th, 2021 (Day 30), leading to the suspension of polymyxin treatment. The kidney injury may have been due to concentration- and time-dependent apoptosis [23]. With plasma concentrations ranging from 1.42 mg/L to 3.87 mg/L, prolonged intravenous administration could be a risk factor for nephrotoxicity in this patient. No obvious neurotoxicity was observed in this study, as the patient was unconscious throughout her ICU stay. However, our proteomics results showed that two well-validated biomarkers for astroglial and neuronal injury, glial fibrillary acidic protein (GFAP) and neurofilament light chain (NfL) [64], were both significantly up-regulated on Day 7. Noticeable skin hyperpigmentation was observed on her face and hands on Day 26, after the commencement of IV polymyxin B. Several recent case studies have reported skin hyperpigmentation associated with IV polymyxin B, with an incidence of approximately 8%–15 % [[65], [66], [67], [68]]. Additionally, increased plasma creatinine (from 24 to 214 μmol/L) and decreasing GFR (from 120.48 to 19.66 mL/[min·1.73 m^2^]) from Day 2 to Day 28 indicated deteriorating renal function, suggesting that intravenous polymyxin B might have been a contributing factor. Consequently, all polymyxin B treatments were paused on Day 30 and replaced by intravenous tigecycline. Unfortunately, the patient died of severe sepsis the following day.

The innate immune system was highly active 24 h after the first ITH/IVT dose of polymyxin B in the ICU. Although we did not examine bacterial responses in the present study, our previous published proteomic work discovered the down-regulation of bacterial iron acquisition systems in *A. baumannii* using a tripartite model involving *A. baumannii* strain AB5075, human leukemia monocytic cell line THP-1 and polymyxin B (30 mg/L, 1 h) [69]. Considering the ferroptosis activation in our CSF proteomics data here, we believe that iron competition played a key role in the immune response to bacterial infection.

Microglia are resident macrophages of the brain which constitute 5–20 % of all cells in the CNS [70]. They play an important role in neuroinflammation, as indicated by the dysregulation of microglia-associated marker proteins (Fig. 5). Translocator protein (TSPO) is expressed on the outer mitochondrial membrane of microglia, with low expression in resting microglia and high expression in activated microglia [71]. Expression of TSPO is particularly high during inflammation [71], making it an excellent biomarker for microglial activation. Twenty-four hours after the first ITH administration of polymyxin B in the ICU, TSPO expression was up-regulated 3.3-fold, followed by down-regulation at Day 7 (1.1-fold) and Day 26 (2.5-fold, significantly). This indicates synergistic antibacterial activity between microglia and polymyxin B at 24 h.

The BBB is the interface between the blood and the brain, protecting central neurons from systemic infection and inflammation [30]. Many pathological conditions can cause BBB dysfunction, including hypoxic and ischemic insults [72], Parkinson's and Alzheimer's diseases [73], brain tumors [74], and systemic infection and inflammation [75]. In the present study, the patient experienced a cerebral hemorrhage on July 27th, 2021, indicating a serious disruption of the BBB. This patient had a long-history hypertension and was diagnosed with a lung infection on July 28th, 2021–both of which are risk factors for BBB breakdown. Therefore, it is likely that the BBB had not fully recovered when ITH polymyxin B was commenced. The patient received the first ITH dose of polymyxin B at ZhuJiang Hospital on August 24th, 2021, and the second on August 28th in the Neurosurgery Department of our hospital. CSF proteomic analysis revealed worsened BBB disruption at 24 h, as evidenced by the down-regulation of junctional adhesion molecules and basement membrane proteins. However, by Day 7 and Day 26, the disruption had reversed, with junctional adhesion molecules and basement membrane proteins up-regulated, indicating BBB repair. Despite this repair, other systems and organs—including the respiratory system, coagulation system, liver, and kidneys—were damaged, ultimately leading to the patient's death from sepsis.

Two factors may contribute to the initial worsening of BBB disruption. Firstly, LPS in the peripheral blood and the ventricle could be a disruptive factor. LPS affects the tight junction integrity of BBB via prostanoids and nitric oxide in a dose-dependent manner [75]. Therefore, polymyxin B could alleviate the BBB disruption by binding with the LPS and decreasing the abundance of LPS by killing bacteria. To verify this assumption, the LPS concentrations in the CSF at 0 h, 24 h, Day 3, Day 7, Day 9 and Day 26 were measured using LAL assay. The concentration of LPS was 0.96 EU/ml at 0 h and 0.8 EU/ml at 24 h, but undectable after Day 3. These results strongly support our assumption that polymyxins help to repair the impaired BBB by reducing the LPS abundance. Second, pro-inflammatory cytokines such as IL-6 and tumor necrosis factor-α (TNF-α) can activate reactive oxygen species (ROS) produced by NADPH oxidases and the JAK/STAT pathway, causing BBB damage [76]. In this study, IL-6 was increased 1.3-fold at 24 h but decreased 1.6-fold at Day 7 and 1.7-fold at Day 26; antioxidant enzymes superoxide-dismutase 3 (SOD3) and catalase (CAT) that inhibiting ROS generation were significantly down-regulated at 24 h but up-regulated at Day 26. NADPH oxidase subunit CYBB was also significantly increased at 24 h but decreased afterwards, and NADPH oxidase subunits NCF1, NCF2 and NCF4 were increased at 24 h but decreased at Day 26. Key proteins in the JAK/STAT pathway (STAT1, STAT3, STAT5A and STAT6) were significantly increased at 24 h, indicating the activation of the JAK/STAT signaling at 24 h. These DEPs results indicated that at 24 h after administration, ITH polymyxin B led to strong antibacterial and pro-inflammatory responses.

The results of this study may help to optimise the clinical use of IVT/IVH polymyxins. For example, even if pathogens are not cultured from the CSF after antimicrobial treatment, a small amount of ITH/IVT polymyxins could be used to alleviate BBB damage in patients with ventriculitis. Timely TDM is also strongly recommended to avoid potential toxicity and ensure the clinical efficacy of polymyxins. There are several limitations associated with this study. Firstly, it involved only a single patient, and additional studies with a larger number of patients with CNS infections are required to better understand CSF responses to polymyxins after ITH administration, although conducting such studies is challenging. The current results were constrained by the patient's physical conditions, and the variability in individual responses to ITH/IVT polymyxins could not be assessed. Second, two doses of ITH polymyxin B were administered before the first CSF sample was obtained in this study. In clinical practice, patients are regularly transferred between hospitals and departments, and a significant proportion of ICU patients receive antibiotics and other treatments before admission. Third, the patient received several other concomitant antibiotics, which may have contributed to changes in protein expression.

## 5 Conclusion

In summary, this is the first CSF proteomic study on polymyxins and revealed the efficacy of ITH/IVT polymyxins for the treatment of CNS infections caused by MDR *A. baumannii*. Our study also provides important mechanistic insights into the immune activation and BBB repairment after ITH/IVT polymyxin administration. Potential neurotoxicity and skin hyperpigmentation induced by polymyxin B highlight the importance of safe use of this last-line class of antibiotics, and timely TDM should be conducted for optimizing their dosing regimens in the clinical practice.

## CRediT authorship contribution statement

**Mengyao Li:** Writing – original draft, Funding acquisition, Formal analysis, Conceptualization. **Dongyu Liu:** Conceptualization. **Phillip J. Bergen:** Writing – review & editing. **Silin Liang:** Data curation. **Juan Chen:** Formal analysis, Data curation. **Zhi Ying Kho:** Formal analysis. **Jing Lu:** Formal analysis. **Huiying Sun:** Data curation. **Weiqing Hong:** Data curation. **Xiaofen Liu:** Formal analysis. **Chengying Hong:** Formal analysis. **Youlian Chen:** Formal analysis. **Wei Li:** Data curation. **Hongxia You:** Data curation. **Shunyao Xu:** Formal analysis. **Yu Wang:** Data curation. **Huaiji Gao:** Visualization. **Chun Hin Lam:** Visualization. **Jian Li:** Writing – review & editing, Supervision, Conceptualization. **Xiaoyin Chen:** Supervision, Funding acquisition. **Xueyan Liu:** Supervision, Funding acquisition.

## Ethics approval

The study was conducted according to the guidelines of the Declaration of Helsinki, and approved by the Ethic Committee of Shenzhen People's Hospital, Shenzhen, China (Approval No. LL-KY-2021620, 25th June 2021) and informed consent was obtained from the patient's family.

## Data availability statement

Proteomic data will be available from the authors upon reasonable requests. Proposals will be reviewed and approved by the authors based on scientific merit.

## Declaration of competing interest

All authors declare no competing interests related to this study.

J.L. received grants, speaking honoraria, and consulting fees from Northern Antibiotics, Avexa, Genentech, Healcare, CTTQ, Aosaikang, Jiayou Medicine, MedCom, Fansheng Biotech, DanDi BioScience, Qpex Biopharma, and Xellia Pharmaceuticals.
