# Supplementary material for: Cerebrospinal fluid proteomics reveals the innate immunity and blood-brain barrier dysregulation in a patient with multidrug-resistant Acinetobacter baumannii ventriculitis treated with intrathecal and intravenous polymyxin B
Source: Heliyon. 2024 Dec 7;10(24):e40893. doi: 10.1016/j.heliyon.2024.e40893 (PMC11699078; doi:10.1016/j.heliyon.2024.e40893)
Supplement: Multimedia component 1 [file mmc1.docx]

**
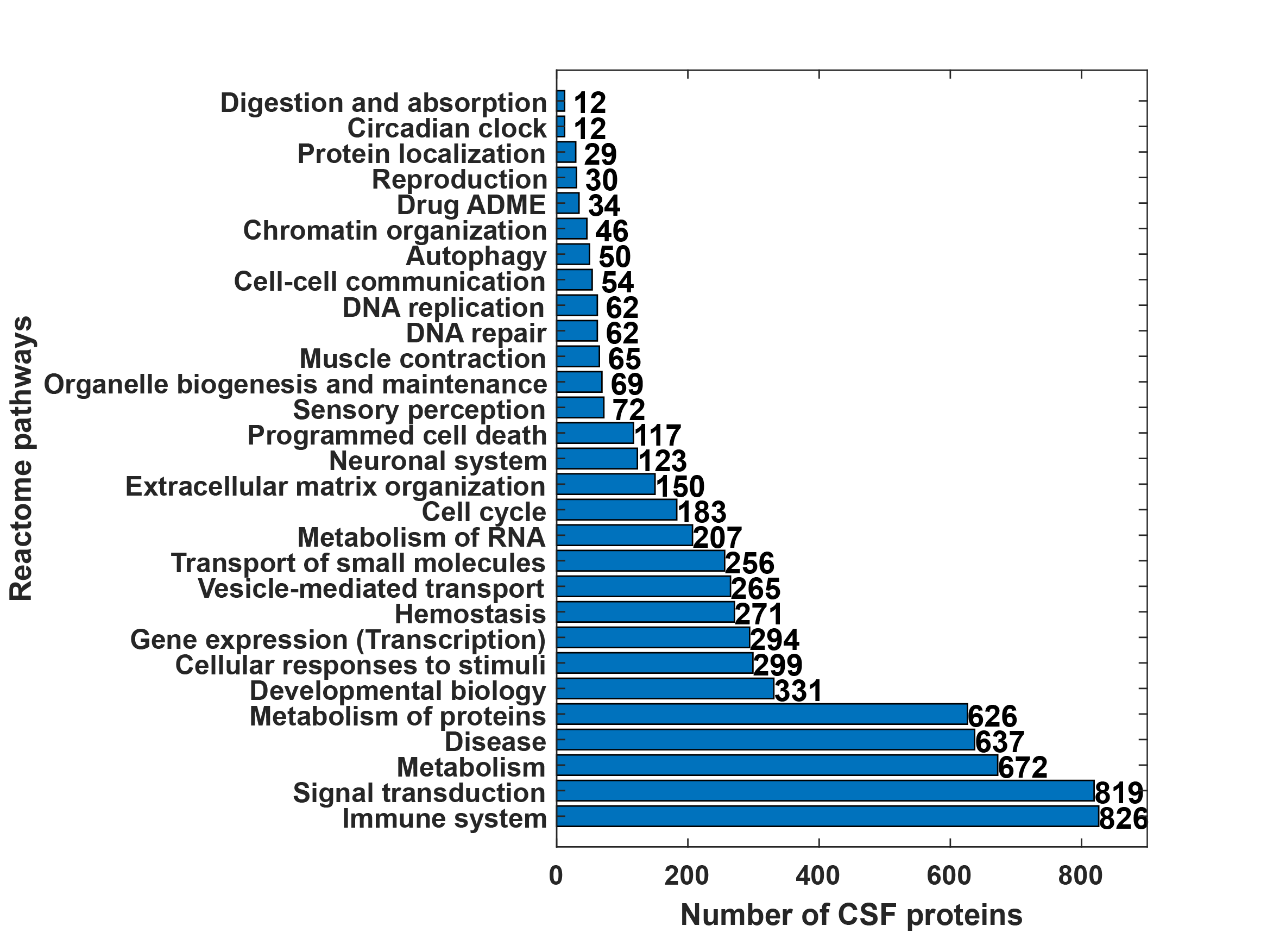
**

**Figure S1**


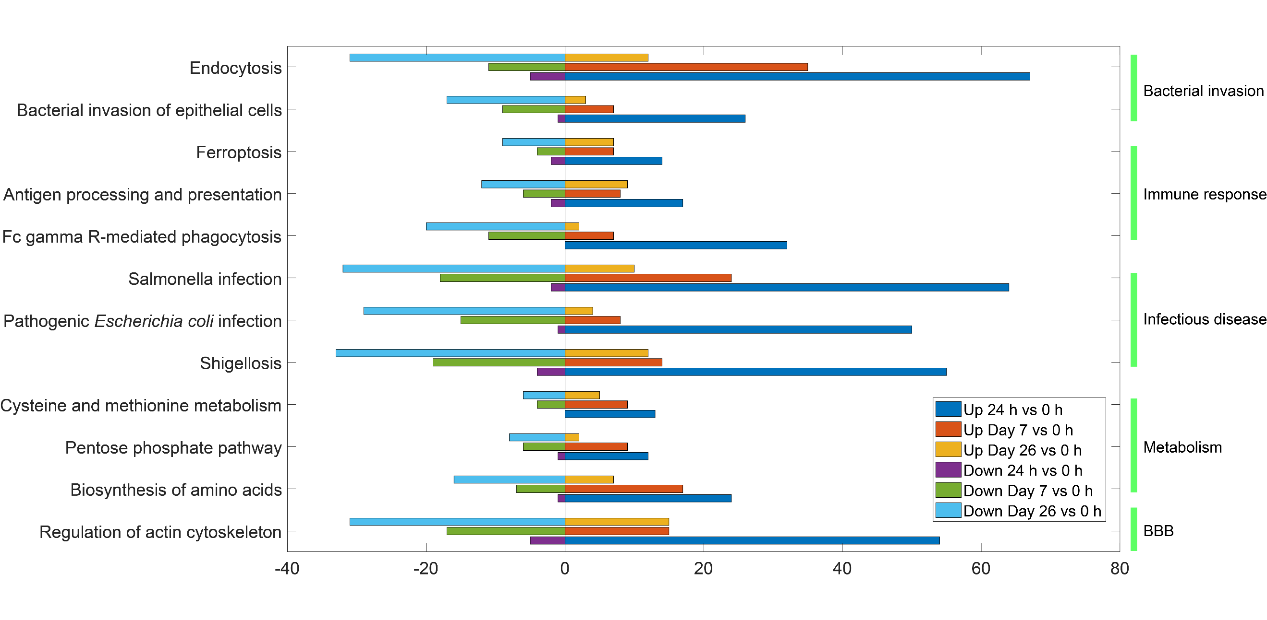


**Figure S2**

**Figure legends**

**Figure S1.** CSF proteins annotated and enriched by Reactome.

**Figure S2.** Common KEGG pathways shared at each of the three time points.
